# In-depth investigation of large axial magnetic anisotropy in monometallic 3d complexes using frequency domain magnetic resonance and *ab initio* methods: a study of trigonal bipyramidal Co(ii)†
†Electronic supplementary information (ESI) available: Synthetic procedures, crystallographic data, powder X-ray diffraction (PXRD) patterns, additional magnetic, EPR and FDMR data, further computational details. CCDC 1891631. For ESI and crystallographic data in CIF or other electronic format see DOI: 10.1039/c9sc00987f


**DOI:** 10.1039/c9sc00987f

**Published:** 2019-05-20

**Authors:** Moya A. Hay, Arup Sarkar, Gavin A. Craig, Lakshmi Bhaskaran, Joscha Nehrkorn, Mykhailo Ozerov, Katie E. R. Marriott, Claire Wilson, Gopalan Rajaraman, Stephen Hill, Mark Murrie

**Affiliations:** a WestCHEM , School of Chemistry , University of Glasgow , University Avenue , Glasgow , G12 8QQ , UK . Email: mark.murrie@glasgow.ac.uk; b Department of Chemistry , Institute of Technology Bombay , Powai , Mumbai , Maharashtra 400 076 , India . Email: rajaraman@chem.iitb.ac.in; c Department of Physics , Florida State University , Tallahassee , FL 32306 , USA . Email: shill@magnet.fsu.edu; d National High Magnetic Field Laboratory , 1800 E. Paul Dirac Drive Tallahassee , FL 32310 , USA; e Max Planck Institute for Chemical Energy Conversion , Stiftstr. 34-36 , 45470 Mülheim an der Ruhr , Germany

## Abstract

The magnetic properties of 3d monometallic complexes can be tuned through geometric control, owing to their synthetic accessibility and relative structural simplicity. Monodentate ligands offer great potential for fine-tuning the coordination environment to engineer both the axial and rhombic zero-field splitting (ZFS) parameters. In [CoCl_3_(DABCO)(HDABCO)] (**1**), the trigonal bipyramidal Co(ii) centre has two bulky axial ligands and three equatorial chloride ligands. An in-depth experimental and theoretical study of **1** reveals a large easy-plane magnetic anisotropy (+ve *D*) with a negligible rhombic zero-field splitting (*E*) due to the strict axial symmetry imposed by the *C*_3_ symmetric ligand and trigonal space group. The large easy-plane magnetic anisotropy (*D* = +44.5 cm^–1^) is directly deduced using high-field EPR and frequency-domain magnetic resonance (FDMR) studies. *Ab initio* calculations reveal a large positive contribution to the *D* term arising from ground state/excited state mixing of the ^4^E′′ states at ∼4085 cm^–1^ and a minor contribution from the spin–flip transition as well. The nature of the slow relaxation in **1** is elucidated through analysis of the rates of relaxation of magnetisation, taking into account Raman and direct spin–lattice relaxation processes and Quantum Tunnelling of the Magnetisation (QTM). The terms relating to the direct process and QTM were found based on the fit of the field-dependence of *τ* at 2 K. Subsequently, these were used as fixed parameters in the fit of the temperature-dependence of *τ* to obtain the Raman terms. This experimental–theoretical investigation provides further insight into the power of FDMR and *ab initio* methods for the thorough investigation of magnetic anisotropy. Thus, these results contribute to design criteria for high magnetic anisotropy systems.

## Abstract 2

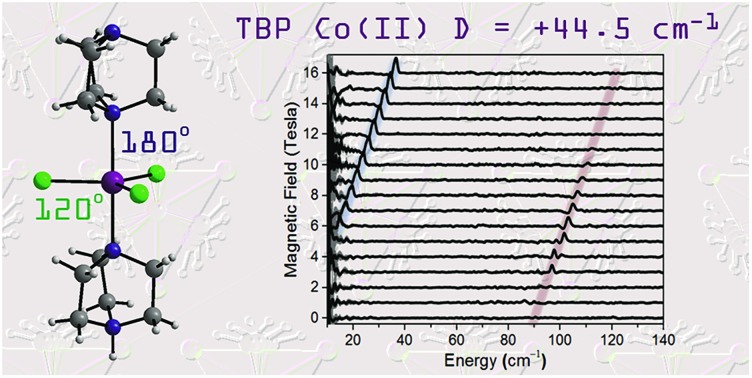
Symmetry control using DABCO generates a large easy-plane magnetic anisotropy with zero rhombic anisotropy.

## Introduction

The investigation of molecules that retain their magnetisation in the absence of an applied magnetic field, single-molecule magnets (SMMs), has been driven by the need to find new materials for high-density data storage and quantum computing.^1^ Recent work in this field has focused on engineering high magnetic anisotropy in complexes containing a single paramagnetic ion, which has led to the first examples of SMMs showing slow relaxation of their magnetisation above the temperature of liquid nitrogen.^2^ This approach requires that we develop an understanding of how molecular geometry can be tailored to achieve large spin–orbit coupling (SOC) contributions to magnetic anisotropy, and how undesired relaxation processes can be controlled.^3^ Hence, monometallic 3d complexes have generated significant interest in this area, with simple modification of the ligands influencing the zero-field splitting parameters and, consequently, the observation of slow relaxation of the magnetisation.^4^ The majority of monometallic 3d SMMs are based on the half-integer spin Co(ii) ion, due to its ability to display slow relaxation of magnetisation in a range of coordination environments. The mechanism for relaxation of magnetisation is governed by the magnetic anisotropy, with the dominant processes contributing to spin reversal being dependent on the magnitudes of both *D* and *E*. Slow magnetic relaxation arising from an Orbach process (*i.e.* relaxation involving transitions to real *m*_s_ states) can occur for systems with small/moderate *D* parameters, irrespective of the sign of *D*. For larger *D* values, Raman (two-phonon) and direct (single-phonon) spin–lattice relaxation pathways should also be considered.^5^ Furthermore, if the rhombic zero-field splitting (ZFS) parameter *E* is zero, as is the case in a high symmetry molecule, then the contribution to the relaxation from quantum tunneling transitions mediated by hyperfine or dipole fields will be further inhibited, thus isolating spin–lattice relaxation processes. These complexes therefore present ideal model systems to study spin–lattice relaxation, which can often also be detrimental in other more complex molecules. To achieve a large *D* value in combination with a zero, or negligible, *E* term requires geometric control of the coordination environment. Herein, we use the bulky *C*_3_ symmetric ligand 1,4-diazabicyclo[2.2.2]octane (DABCO) to achieve trigonal symmetry around the Co(ii) ion in the monometallic complex [Co^II^Cl_3_(DABCO)(HDABCO)] (**1**). Strict *D*_3h_ symmetry, in the first coordination sphere, is imposed by the molecules packing in the *R*32 space group.^6^ We use a combination of magnetic susceptibility measurements, *ab initio* calculations, high field electron paramagnetic resonance (HF-EPR), and frequency-domain magnetic resonance (FDMR) spectroscopy to elucidate the relaxation mechanisms and the large easy-plane magnetic anisotropy for Co(ii) in a strict trigonal bipyramidal (TBP) coordination environment.

## Results and discussion

### Structure

**Fig. 1:**
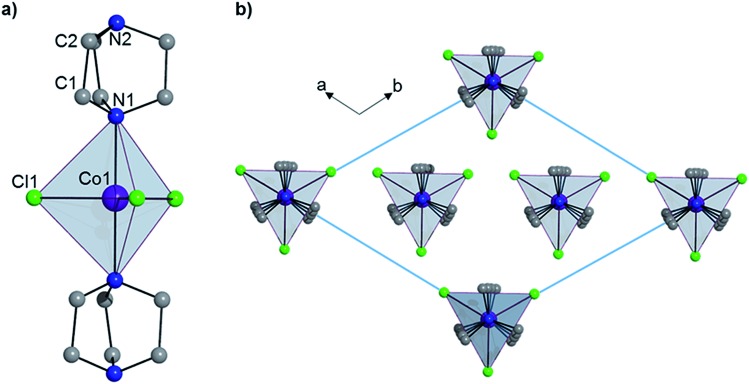
(a) The structure of **1** and (b) the crystal packing as viewed along the *c*-axis. Hydrogen atoms have been omitted for clarity.

Complex **1** crystallizes in the trigonal *R*32 space group (see Table S1†) and comprises a central Co(ii) ion in a trigonal bipyramidal coordination environment (*D*_3h_) with two axial DABCO ligands and three equatorial chloride ligands (see Fig. 1). Charge balance requires one cationic HDABCO^+^ and one DABCO ligand per molecule and, crystallographically, the proton on N2 is modeled with half-occupancy. The Co–Cl bond distance is 2.3252(8) Å, the Co–N distance is 2.2680(5) Å, and the axial and equatorial bond angles are 180° and 120°, respectively. Continuous shape measures (CShMs) were calculated using the program SHAPE to quantify the degree of distortion around the Co^II^ center from the ideal trigonal bipyramidal coordination geometry,^7^ and the value of 0.015 obtained for **1** indicates an almost perfect TBP environment. The shortest intermolecular Co···Co distance is 7.3627(13) Å and propagates along the *a*- and *b*-axes of the crystal lattice. There is a strong intermolecular hydrogen-bonding interaction between neighbouring molecules of **1** (N2–H···N2′ with an N2···N2′ distance of 2.638(10) Å), which pack into a 1D chain along the 3-fold symmetry axis of each molecule (N1–Co1–N1′), which coincides with the crystallographic *c*-axis (see Fig. 1).

### Theoretical studies

**Fig. 2:**
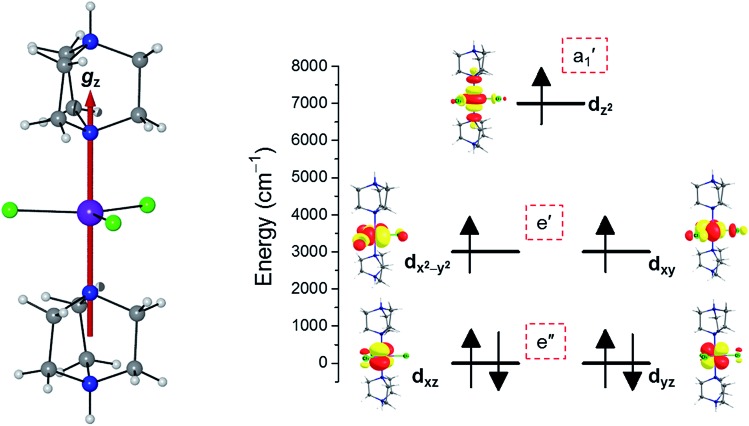
Crystal structure of complex **1** along with the orientation of the *z*-axis shown in red, which coincides with the *C*_3_ axis of the molecule and the *c*-axis of the crystal lattice (left); NEVPT2-LFT d-orbital energy diagram of the Co(ii) ion in **1** (right).

To predict the likely nature of the ZFS for complex **1**, *ab initio* calculations were performed. Using the NEVTP2 method on top of the CASSCF wave-function, a large positive value of *D* = +44.2 cm^–1^ is obtained (*vide infra* for computational details).^8^ This is unusual given the majority of TBP Co(ii) complexes reported in the literature have small negative *D* values (see Table S4 in ESI†). However, it is consistent with the nature of the coordinated ligands with strong π donating character in the equatorial positions (Cl^–^) and weak σ donating character in the axial positions ([DABCO]^+^).^9^ The major anisotropy axis (associated with *g*_*zz*_ and *D*_*zz*_) is collinear with the *C*_3_ rotational axis imposed by the crystallographic space group (Fig. 2). The origin of the easy plane anisotropy in **1**, which yields the positive value of *D*, can be rationalized by using the spin-allowed part of the second-order perturbative equation (see eqn (3) in Computational details). The ground state electronic configuration of the d^7^ Co(ii) ion corresponds to a non-degenerate 
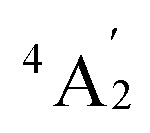
 term with respect to *D*_3h_ point group symmetry. Excitation of a single electron from the degenerate d_*xz*_ or d_*yz*_ orbitals to the d_*x*^2^–*y*^2^_ or d_*xy*_ orbitals (Fig. 2) yields four excited electronic states: 
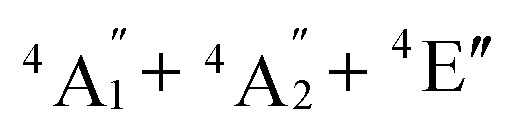
 (Fig. S3†). Each electronic state consists of multiple contributions from different d electron configurations (see Table S3, ESI†). The major contribution to *D* arises from ground state/excited state mixing of the ^4^E′′ states at ∼4085 cm^–1^. The calculations predict that the two ^4^E′′ states are almost degenerate, but split by ∼3 cm^–1^ (Table S3, ESI†). Hence, eqn (2) yields a finite *E*/*D* ratio of 0.000473. However, the computational method should not be considered accurate to four decimal places, and the *E*/*D* ratio is expected to be exactly zero (and the ^4^E′′ states degenerate) due to the local *D*_3h_ point group symmetry found in crystals of **1**. There is another smaller, but non-negligible positive contribution to *D* (+12 cm^–1^) arising from the excited 
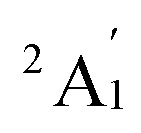
 state which lies ∼19 600 cm^–1^ above the ground state (Table S3, ESI†). Importantly, it should be noted that the overall/final *D* value is obtained directly from the Effective Hamiltonian Approach (EHA)^20^ after diagonalisation of the whole *D* matrix, including 10 quartets and 40 doublets, and hence the final obtained *D* value of 44.2 cm^–1^ does not reflect the sum of individual *D* values arising from various electronic states. Additionally, *ab initio* calculations yield *g*-tensor components for the *S* = 3/2 manifold of *g*_*zz*_ = 1.988 and *g*_*xx*_ = *g*_*yy*_ = 2.433 or, alternatively, effective values of *g*eff*zz* = 1.989 and *g*eff*xx* = *g*eff*yy* = 4.860 for the lowest Kramers doublet. These estimates are in very good agreement with the values obtained from experiments (see below). The computed identical *g*_*xx*_ and *g*_*yy*_ values are consistent with the negligible rhombicity present in the complex.

### EPR and FDMR measurements

**Fig. 3:**
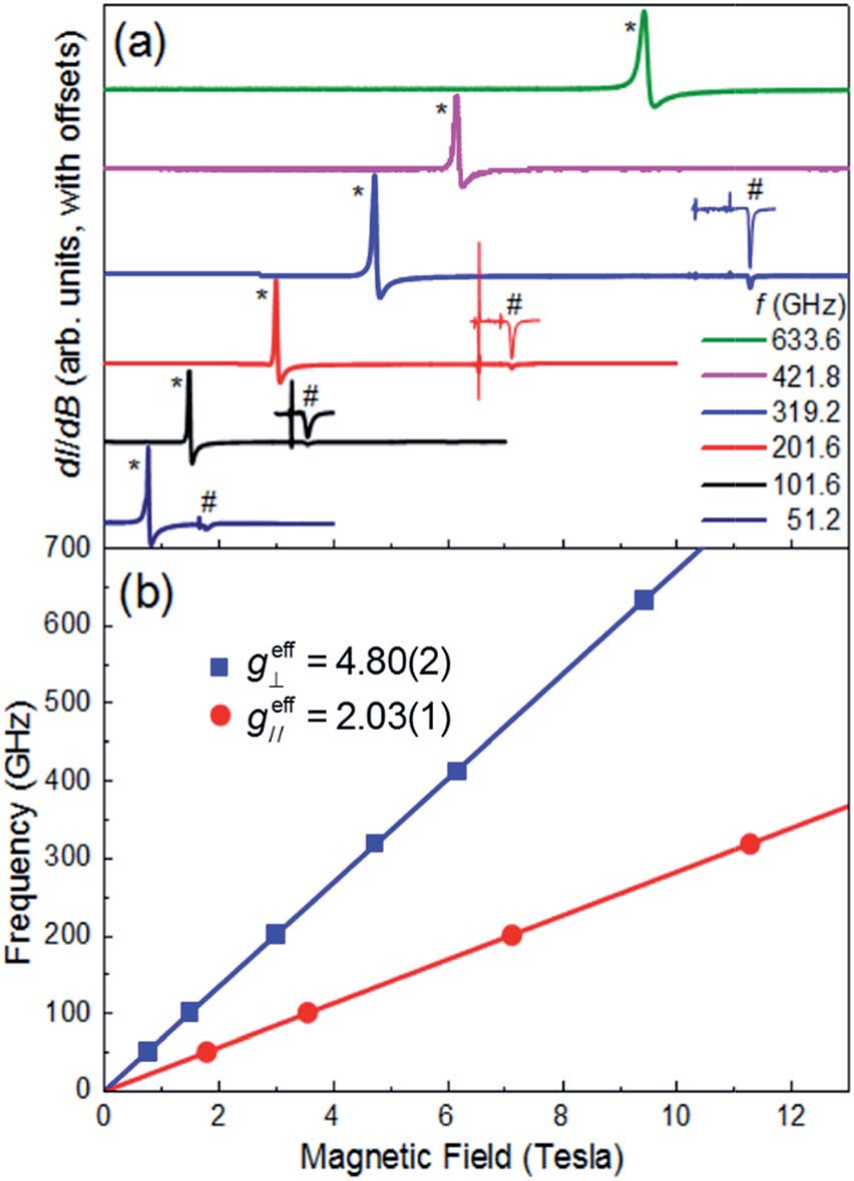
(a) Multi-frequency powder EPR spectra for **1**, recorded at *T* = 5 K in field derivative, d*I*/d*B*, mode (where *I* represents the absorption intensity and *B* the magnetic field strength); the frequencies are given in the legend. Features attributed to the perpendicular and parallel components of the spectra are marked with * and #, respectively. The parallel portions of the spectra have been expanded for three of the frequencies (101.6, 201.6 and 319.2 GHz) in order to ease viewing (see ESI† for explanation of the sharp features just below the parallel components of the spectra). (b) Frequency *versus* resonance position plot corresponding to the perpendicular and parallel mode excitations seen in (a). The solid lines are linear fits to the data (with the zero-field offsets constrained to the origin) from which the effective *g*-values are obtained (see ESI† for further details).

Multi-high-frequency EPR measurements were performed on a microcrystalline powder sample of **1** in order to provide spectroscopic insights into its magnetic anisotropy (Fig. 3a). The spectra reveal two sharp features that can be attributed to parallel and perpendicular excitations within the lowest Kramers doublet, with effective Landé factors *g*eff∥ = 2.03(1) & *g*eff⊥ = 4.80(2) (Fig. 3b). In the limit *hf* ≪ *Δ*_K_, where *Δ*_K_ [= 2(*D*^2^ + 3*E*^2^)^1/2^] is the zero-field gap between Kramers doublets, *f* is the microwave frequency and *h* the Planck constant, *g*eff∥ & *g*eff⊥ are insensitive to the absolute values of the ZFS parameters *D* and *E*. However, a finite *E* parameter is expected to give rise to a splitting of the perpendicular component of the spectrum, with a magnitude that depends on the ratio of *E*/*D*. The absence of such a splitting gives an upper bound of *E*/*D* ≤ 0.006 (see ESI†), consistent with the theoretical studies. Setting *E*/*D* = 0, which is a very good approximation in this case, allows us to directly relate the effective *g*-factors associated with the lowest Kramers doublet to those of the full *S* = 3/2 state, giving *g*_*zz*_ = 2.03(1) and *g*_*xx*_ = *g*_*yy*_ = 2.40(1), in good agreement with the theoretical studies, and again confirming the easy-plane anisotropy (see ESI† for detailed explanation). We note that there is no physically realistic scenario under which the obtained effective *g*-factors can be rationalized on the basis of an easy-axis anisotropy.

**Fig. 4:**
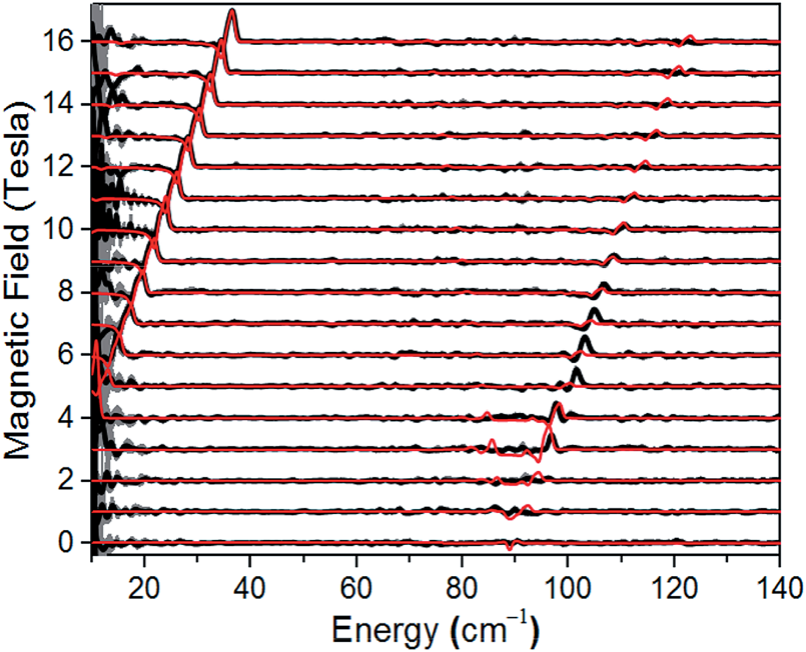
Normalized experimental FDMR spectra (black) with simulations (red) superimposed; see main text and ESI† for parameters and explanation of the analysis. The data were recorded at a temperature of 4.2 K, and the baseline of each spectrum is positioned according to the applied field strength on the ordinate. The gray shading provides a measure of the energy-dependent standard deviation of the FDMR signal from four separately recorded spectra.

A direct measure of *Δ*_K_ and, hence, *D* requires magneto-optical measurements at far higher frequencies. Fig. 4 displays normalized frequency-domain magnetic resonance (FDMR) spectra recorded at a temperature of 4.2 K for a powder sample of **1**, spanning the range from 10 to 140 cm^–1^ (see also Fig. S5 and ESI† for further details of the data analysis). As can clearly be seen, resonances are apparent in two regions of the figure: a low frequency branch below 40 cm^–1^, which extrapolates to zero energy at zero field; and a high-frequency branch centered at ≈89 cm^–1^ at zero field. The former corresponds simply to the perpendicular (*g*eff⊥) resonance associated with the lowest Kramers doublet, and is observed in the low-frequency EPR experiments (Fig. 3a). Meanwhile, the high frequency branch corresponds to an allowed inter-Kramers transition. Consequently, the zero-field transition frequency (see also Fig. S6†) corresponds exactly to *Δ*_K_ = 2*D* (assuming negligible *E*). Superimposed on the FDMR spectra in Fig. 4 are optimum simulations that assume *D* = +44.5 cm^–1^, and exactly the same *g*-values deduced on the basis of the EPR measurements. The obtained *D* value, which is the only adjustable parameter in the simulations (apart from a normalization factor needed to reproduce the strengths of the FDMR signals), is in excellent agreement with the *ab initio* calculations and magnetic measurements (*vide infra*).

### Magnetic properties

**Fig. 5:**
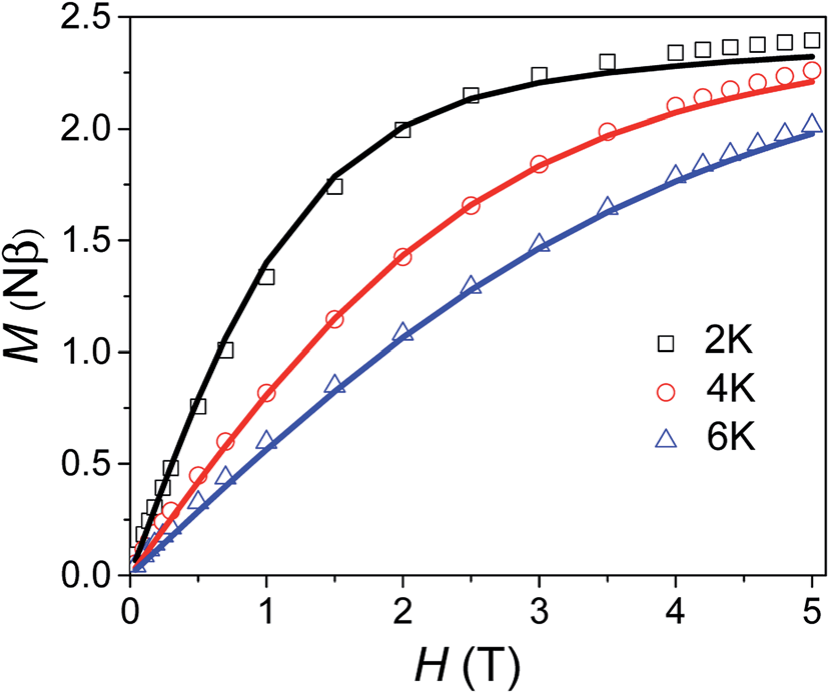
Variable field magnetisation data for **1** collected at 2, 4 and 6 K between 0 to 5 T. The solid line corresponds to the fit (see text for details).

Variable field magnetisation data (*M vs. H*) of **1** are shown in Fig. 5, with the temperature dependence of the molar magnetic susceptibility product (*χ*_m_*T vs. T*) presented in Fig. S7 in the ESI.† The *χ*_m_*T vs. T* and *M vs. H* data were fitted simultaneously using the program Phi (the Hamiltonian used and details of the fit are presented in the SI†).^10^ Considering the results of the FDMR measurements (*vide supra*), the axial ZFS parameter was fixed at *D* = +44.5 cm^–1^. Additionally, the approximation that *E* = 0 for an axial system was applied in the case of **1**, consistent with the HF-EPR measurements and *ab initio* calculations. Although the calculations and HF-EPR measurements indicated an anisotropic Landé tensor, the magnetic data can be fitted using an isotropic *g*-factor: *g* = 2.55 was obtained from the fit, very close to the value estimated from the experimental *χ*_m_*T* value at 290 K (*cf.* 2.58 cm^3^ mol^–1^ K). In comparison to previously reported TBP Co(ii) complexes which employ polydentate ligands, |*D*| is much larger for **1** (see Table S4†).4c,^9^,^11^ In only one case has a higher |*D*| value been reported for a Co(ii) TBP coordination polymer, with the parameters *D* = +59 cm^–1^, *E* = 7 cm^–1^ and *g* = 2.36.^12^ However, these parameters were obtained only from a fit of the magnetic data, and have not been confirmed by spectroscopic measurements or theoretical studies. The key point for **1** is that not only is *D* very large for TBP Co(ii), but that the rhombicity is zero due to the strict axial symmetry imposed by the combination of the three-fold symmetric DABCO ligands and the *R*32 space group.

**Fig. 6:**
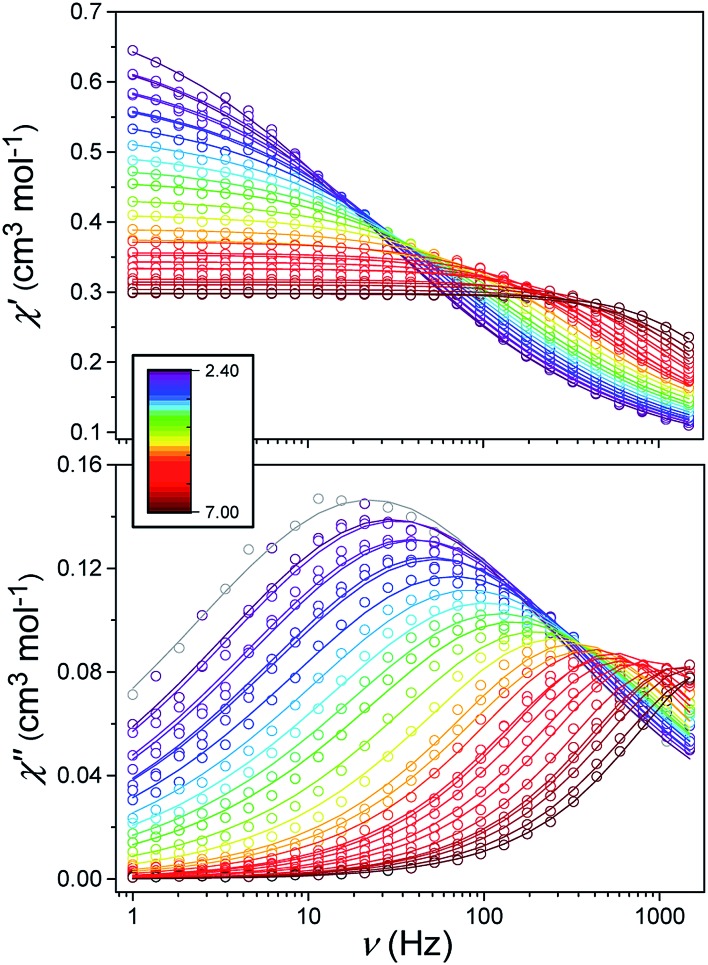
Frequency-dependence of the in-phase (*χ*′) and out-of-phase (*χ*′′) susceptibility signal over a range of temperatures and conducted under *H*_dc_ = 2500 Oe.

**Fig. 7:**
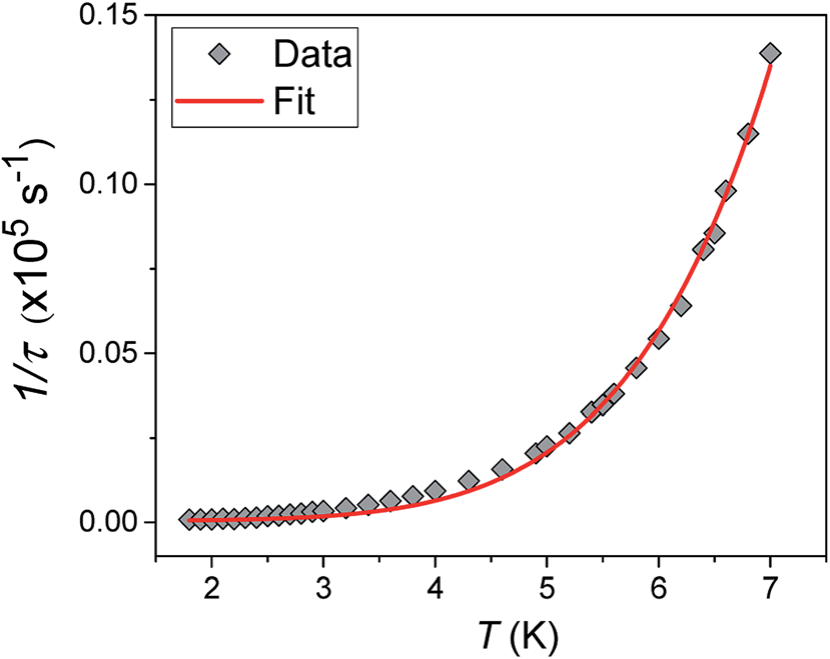
1/*τ vs. T* with the optimised fit to eqn (1) considering Raman, direct and QTM processes shown as a solid red line (see text for details).

Alternating current (ac) susceptibility measurements were carried out to investigate the relaxation dynamics of **1**. In zero applied dc field, no out-of-phase (*χ*′′) response was observed, but on applying a dc field, a *χ*′′ is observed (see Fig. S8–S11†). The field dependence of this slow relaxation of magnetisation in **1** showed an optimum response under an applied dc field of 2500 Oe (Fig. S10†), and so the frequency dependence of the out-of-phase component to the ac susceptibility, *χ*′′, was measured over the temperature range 2.4 to 7.0 K under this field (see Fig. 6 and S11†). The maxima in *χ*′′ move out of the accessible frequency range of the SQUID above 6.6 K. For **1**, a fit of the relaxation data to an Arrhenius law, corresponding to a two-phonon Orbach process, should yield a value for the activation energy of ∼|2*D*|. Attempts to fit the data in this way failed in two respects: firstly, they did not fit at all well to an Arrhenius behavior; second, the fit yielded an activation energy that is much smaller than |2*D*| (21.5 cm^–1^*vs.* 89 cm^–1^, see Fig. S13 in the ESI†). Subsequently, a fit of 1/*τ vs. T* (see Fig. 7) was performed using the extended Debye model shown in eqn (1), which takes into account the Raman (*τ* ∝ *T*^*n*^) and direct (*τ* ∝ *T*) spin–lattice relaxation processes and Quantum Tunnelling of the Magnetisation (QTM), given as the 1st, 2nd and 3rd terms, respectively.^13^ The Raman process does dominate, although inclusion of a direct process is necessary to fit the data below 3 K. To avoid over-parameterisation, the terms relating to the direct process (*A*) and QTM (*B*_1_ and *B*_2_) were fixed based on fits of the field-dependence of *τ* at 2 K between 200 and 4000 Oe (see Fig. S12 and eqn (S6)†) yielding *A* = 277.9 (20.9) s^–1^ Oe^–2^ K^–1^; *B*_1_ = 302.4 (9.5) s^–1^ and *B*_2_ = 181.2 (17.2) Oe^–2^. For the Raman process we then obtain *C* = 0.20(0.04) s^–1^ K^–*n*^ with *n* = 5.70(0.09), where *n* = 9 is expected for a Kramers ion, although this value may be lower if optical and acoustic phonons are taken in to account.^5^,^14^
1
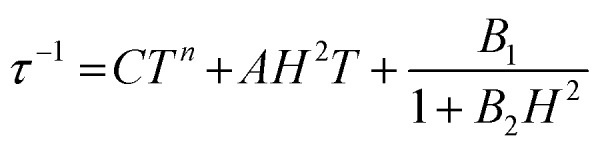



## Conclusions

We have demonstrated a strategy for generating a large easy-plane magnetic anisotropy in trigonal bipyramidal Co(ii). The enhanced magnetic anisotropy is confirmed by high field EPR, frequency-domain magnetic resonance (*D* = +44.5 cm^–1^) studies and *ab initio* methods. Compared to other trigonal bipyramidal complexes, such as those using tetradentate ligands, we show that a substantial increase in the axial magnetic anisotropy results from using a blend of axial/equatorial monodentate ligands. Importantly, the rhombicity in this large positive *D* system, **1**, is zero due to the strict axial symmetry imposed by the combination of *C*_3_-symmetric axial DABCO ligands and crystallisation in a trigonal space group. Complex **1** displays slow relaxation of magnetisation under an applied dc field and the relaxation data can be accounted for by considering Raman and direct processes along with Quantum Tunnelling of the Magnetisation. Future work will focus on related systems that will allow us to probe further the role of spin-phonon interactions and, subsequently, how these can be controlled through external stimuli such as high pressure. We will also consider **1** and related species as monometallic building blocks to design larger spin systems with significant axial magnetic anisotropy, while keeping the rhombic anisotropy to zero. Such building blocks could be linked into chains or networks, whilst maintaining their symmetry and rigidity. Of particular interest is targeting DABCO radical ligands to link together such moieties to promote strong superexchange interactions, which could be switched on or off in a controlled way using optical or redox probes. Overall, this study contributes to the design criteria for high axial anisotropy 3d monometallic species, to the identification of model systems for the investigation of spin-phonon interactions, and for targets for incorporation into larger functional materials, that can influence the design of future molecular magnetic materials.

## Experimental

### Synthesis

All reagents and solvents were obtained commercially and used without any further purification. Complex **1** was synthesized following a modification of the previously reported procedure.^6^ [CoCl_3_(DABCO)(HDABCO)] (**1**): to a dark purple solution of CoCl_2_ (1.30 g, 4 mmol) in methanol (20 ml) a colorless solution of DABCO (1.12 g, 4 mmol) in methanol (20 ml) was added and stirred for 2 hours at 60 °C. The resultant blue suspension was allowed to cool to room temperature before filtering to yield a pink solution and blue precipitate (the precipitate was discarded as we were unable to obtain a pure crystalline sample from it on re-dissolving). Bright blue single crystals suitable for X-ray diffraction were obtained after one day through vapor–liquid diffusion of the light pink solution with diethyl ether. Yield: 11% (185 mg). IR (*ν* in cm^–1^): 2891 (w), 1470 (s), 1319 (m), 1290 (w), 1180 (w), 1051 (s), 1015 (s), 849 (m), 781 (m), 673 (m). EA analysis: (C_12_H_25_Cl_3_CoN_4_) [%], found: C 36.65, H 6.32, N 13.88; calc: C 36.90, H 6.45, N 14.34.

### Physical methods

Elemental analysis was performed in-house by the microanalysis services at the School of Chemistry, University of Glasgow. IR spectra were collected using a Shimadzu FTIR spectrometer in the range 4000–600 cm^–1^. Crystallographic data were collected for **1** at 100 K using a Bruker APEXII CCD diffractometer with an Oxford Cryosystems *n*-Helix low-temperature device mounted on a sealed tube generator. Structures were solved using SHELXT and refined using full-matrix least-squares refinement using Olex^2^ software.^15^ The powder X-ray pattern was collected on a PANalytical XPert MPD, with Cu Kα1 radiation at ambient temperature over a range of 5° < 2*θ* < 50° using a step size of 0.0167°. The calculated pattern was generated from Mercury using the CIF of the crystal structure at 100 K.^16^ All magnetic measurements were carried out on powdered crystalline samples restrained in eicosane using a Quantum Design MPMS-XL SQUID magnetometer. Data were corrected for the diamagnetic contribution of the sample holder and eicosane by measurements, and for the diamagnetism of the compound (*χ*_m_(dia) = –216 × 10^–6^ cm^3^ mol^–1^). High-field/frequency EPR spectra were collected on a microcrystalline powder sample of **1**, which was immobilized in a polyethylene cup with a Teflon® stopper. The transmission-type spectrometer used in this study employed a 17 T superconducting magnet.^17^ Microwave frequencies were generated in the 50 to 635 GHz range using a phase-locked Virginia Diodes source combined with a series of frequency multipliers. The field modulated EPR signal was obtained *via* lock-in detection using an InSb hot-electron bolometer (QMC Ltd., Cardiff, U.K.). Temperature control was achieved using an Oxford Instruments (Oxford, U.K.) continuous-flow cryostat. FDMR spectra were obtained by recording far-infrared (FIR) spectra under various external magnetic fields.^18^ For this purpose a Bruker Vertex 80v vacuum Fourier transform infrared (FTIR) spectrometer with a resolution of 0.12 cm^–1^ was used. The sample was mounted in a 17 T superconducting magnet with optical access, such that the applied field was parallel to the direction of light propagation (Faraday geometry). The sample was in thermal equilibrium with the liquid helium bath of the magnet and, therefore, at a temperature of 4.2 K. The transmitted FIR radiation was detected using a composite Si bolometer placed directly beneath the sample.

### Computational details

First principle calculations have been carried out using the ORCA 4.0.0. program.^19^ To determine the zero-field splitting (ZFS) Hamiltonian parameters we have employed a multi-configurational *ab initio* (CASSCF/NEVPT2) approach, which is one of the best methods to determine these properties. For the complete active space self-consistent field (CASSCF), all of the 10 quartets and 40 doublets have been considered with an active space consisting of CAS(7,5). During this calculation, the ZORA-def2-TZVP basis set for all elements were considered, which are relativistic contracted versions of def2 basis sets available in ORCA. Also the ZORA (zeroth-order regular approximation) Hamiltonian was employed to account for the scalar relativistic effect. NEVPT2 (N-electron valence perturbation theory second order) was also performed on top of the CASSCF wavefunction to add the dynamic electron correlation effect into the results. Final spin-Hamiltonian parameters and spin–orbit properties have been derived from the QDPT-EHA (quasi-degenerate perturbation theory-effective Hamiltonian approach) method.^20^ This methodology has been successfully used to estimate zero-field splitting in many transition metal complexes.^21^

The zero-field splitting (ZFS) parameters were determined in accordance with the Hamiltonian shown in eqn (2) where the first and second terms describe the axial and rhombic ZFS interactions parameterized through *D* and *E* respectively. The third term takes into account the Zeeman interaction with the spin operator *ŝ*, applied field *B[combining right harpoon above]*, and Landé tensor 
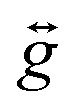
:2
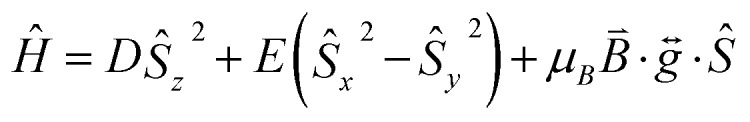



For this complex, the spin-allowed part of the second-order perturbative treatment for the 
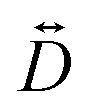
 tensor components are given by eqn (3):3
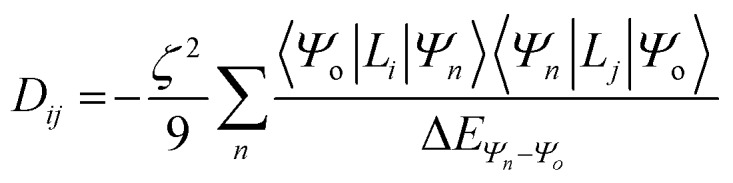
where the sum is taken over excited electronic configurations, *Ψ*_*n*_, with *Ψ*_o_ representing the orbitally non-degenerate 
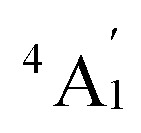
 ground term. *L* is the orbital angular momentum operator, *i*, *j* are the matrix indices and *ζ* is the effective spin–orbit coupling constant of the molecule.

## Conflicts of interest

There are no conflicts to declare.

## Supplementary Material

Supplementary information

Crystal structure data

## References

[cit1] Vignesh K. R., Soncini A., Langley S. K., Wernsdorfer W., Murray K. S., Rajaraman G. (2017). Nat. Commun..

[cit2] Chen Y. C., Liu J. L., Ungur L., Liu J., Li Q. W., Wang L. F., Ni Z. P., Chibotaru L. F., Chen X. M., Tong M. L. (2016). J. Am. Chem. Soc..

[cit3] Lunghi A., Totti F., Sessoli R., Sanvito S. (2017). Nat. Commun..

[cit4] Bar A. K., Pichon C., Sutter J. P. (2016). Coord. Chem. Rev..

[cit5] Gómez-Coca S., Urtizberea A., Cremades E., Alonso P. J., Camón A., Ruiz E., Luis F. (2014). Nat. Commun..

[cit6] Pritchard R. G., Ali M., Munim A., Uddin A. (2006). Acta Crystallogr., Sect. C: Cryst. Struct. Commun..

[cit7] Pinsky M., Avnir D. (1998). Inorg. Chem..

[cit8] Cahier B., Maurice R., Bolvin H., Mallah T., Guihéry N. (2016). Magnetochemistry.

[cit9] Shao F., Cahier B., Guihery N., Riviere E., Guillot R., Barra A.-L., Lan Y., Wernsdorfer W., Campbell V. E., Mallah T. (2015). Chem. Commun..

[cit10] Chilton N. F., Anderson R. P., Turner L. D., Soncini A., Murray K. S. (2013). J. Comput. Chem..

[cit11] Schweinfurth D., Sommer M. G., Atanasov M., Demeshko S., Hohloch S., Meyer F., Neese F., Sarkar B. (2015). J. Am. Chem. Soc..

[cit12] Hou X., Wang X., Liu X., Wang J.-J., Tang L., Ju P. (2018). New J. Chem..

[cit13] (a) Carlin R. L., Magnetochemistry, Springer-Verlag, Berlin, 1986.

[cit14] Colacio E., Ruiz J., Ruiz E., Cremades E., Krzystek J., Carretta S., Cano J., Guidi T., Wernsdorfer W., Brechin E. K. (2013). Angew. Chem., Int. Ed..

[cit15] Dolomanov O. V., Bourhis L. J., Gildea R. J., Howard J. A. K., Puschmann H. (2009). J. Appl. Crystallogr..

[cit16] Macrae C. F., Edgington P. R., McCabe P., Pidcock E., Shields G. P., Taylor R., Towler M., Van De Streek J. (2006). J. Appl. Crystallogr..

[cit17] Hassan A. K., Pardi L. A., Krzystek J., Sienkiewicz A., Goy P., Rohrer M., Brunel L. C. (2000). J. Magn. Reson..

[cit18] Ludwig J., Vasilyev Y. B., Mikhailov N. N., Poumirol J. M., Jiang Z., Vafek O., Smirnov D. (2014). Phys. Rev. B.

[cit19] Neese F. (2018). Wiley Interdiscip. Rev.: Comput. Mol. Sci..

[cit20] Maurice R., Bastardis R., de Graaf C., Suaud N., Mallah T., Guihéry N. (2009). J. Chem. Theory Comput..

[cit21] Singh S. K., Rajaraman G. (2016). Nat. Commun..

