# Supplementary material for: In-depth investigation of large axial magnetic anisotropy in monometallic 3d complexes using frequency domain magnetic resonance and ab initio methods: a study of trigonal bipyramidal Co(ii)
Source: Chem Sci. 2019 May 20;10(25):6354–61. doi: 10.1039/c9sc00987f (PMC6601423; doi:10.1039/c9sc00987f)
Supplement: Supplementary file 1 [file SC-010-C9SC00987F-s001.pdf]

Electronic Supplementary Material (ESI) for Chemical Science.
This journal is © The Royal Society of Chemistry 2019

Supporting Information

In-depth investigation of large axial magnetic anisotropy in monometallic 3*d* complexes using frequency domain magnetic resonance and ab initio methods: a study of trigonal bipyramidal Co(II)

Moya. A. Hay,[a] Gavin A. Craig,[a†] Arup Sarkar,[b], Lakshmi Bhaskaran,[c,d] Joscha Nehrkorn,[d,e] Mykhailo Ozerov,[d] Katie E. R. Marriott,[a] Claire Wilson,[a] Gopalan Rajaraman,[b,*] Stephen Hill,[c,d,*] Mark Murrie[a,*]

[a] WestCHEM, School of Chemistry, University of Glasgow, University Avenue, Glasgow, G12 8QQ (UK).

[b] Department of Chemistry, Institute of Technology Bombay, Powai, Mumbai, Maharashtra, 400 076 (India).

[c] Department of Physics, Florida State University, Tallahassee, FL 32306 (USA)

[d] National High Magnetic Field Laboratory, 1800 E. Paul Dirac Drive Tallahassee, FL 32310 (USA)

[e] Max Planck Institute for Chemical Energy Conversion, Stiftstr. 34-36, 45470 Mülheim an der Ruhr, Germany

[†] Current address: Institute for Integrated Cell-Material Science (WPI-iCeMS), Kyoto University, Yoshida, Sakyo-ku, Kyoto 606-8501 (Japan).

mark.murrie@glasgow.ac.uk

shill@magnet.fsu.edu

rajaraman@chem.iitb.ac.in

Contents

Table S1 Crystal Data and Structure Refinement Parameters. 1

Figure S1 Depiction of crystalline packing of **1**. 2

Table S2 Results from SHAPE studies for complex **1**. 2

Figure S2 Powder X-ray diffraction (PXRD) studies. 3

Table S3 NEVPT2 computed electronic states. 3

Figure S3 NEVPT2 computed quantitative quartet ligand field states. 4

Figure S4 Representation of the components of the ground and excited states 4

High-field EPR Details 5

Frequency Domain Magnetic Resonance Details 7

Figure S5 Normalized experimental FDMR spectra. 8

Figure S6 Normalized experimental FDMR spectra of a thinner sample 9

Figure S7 Variable-temperature magnetic susceptibility 10

Fitting procedure for the dc magnetic data 10

Table S4 Previously reported zero-field splitting (ZFS) parameters for a selection of monometallic Co(II) complexes with TBP geometry. 11

Figure S8 Field dependence of the ac susceptibility. 12

Figure S9 Argand diagram of the field dependence of the ac susceptibility 12

Figure S10 Field dependence of the relaxation rates 13

Figure S11 Argand plots of the temperature-dependence of the ac susceptibility 13

Figure S12 Field dependence of the inverse of the relaxation rates (1/τ) 14

Fitting procedure for the temperature dependent ac magnetic data 14

Table S5 Cole-Cole fit value for 1 15

Figure S13 Arrhenius plot of the temperature-dependence of the relaxation rates. 16

Table S6 Fitting parameters for Raman and direct processes for complex **1** 16

References 17

Table S1 Crystal Data and Structure Refinement Parameters.

	**1**
Empirical formula	$C_{12}H_{25}N_4Cl_3Co$
Formula weight	390.64
Temperature (K)	100(2)
Crystal system	trigonal
Space group	R32
a, *b* (Å)	10.581(3)
c (Å)	12.329(3)
α, *β* (°)	90
γ (°)	120
Volume (Å^3)	1195.4(6)
Z	3
ρ_{calc} (g/cm^3)	1.628
μ (mm^{-1})	1.575
F(000)	609.0
Crystal size (mm^3)	0.3 × 0.05 × 0.05
Radiation	MoKα (λ = 0.71075)
2Θ range for data collection (°)	5.54 to 54.884
Index ranges	$-13 \leq h \leq 9$, $-12 \leq k \leq 13$, $-15 \leq l \leq 15$
Reflections collected	5214
Independent reflections	607 [R_{int} = 0.0875, R_{sigma} = 0.0558]
Data/restraints/parameters	607/0/33
Goodness-of-fit on F^2	1.105
Final R indexes [I>=2σ (I)]	R_1 = 0.0376, wR_2 = 0.0644
Final R indexes [all data]	R_1 = 0.0453, wR_2 = 0.0674
Largest diff. peak/hole (e Å^{-3})	0.49/-0.42
Flack parameter	0.02(4)

Figure S1 Depiction of crystalline packing of **1** with axes indicated. As depicted, purple = cobalt; green = chlorine; blue = nitrogen; grey = carbon; hydrogen atoms have been omitted for clarity.

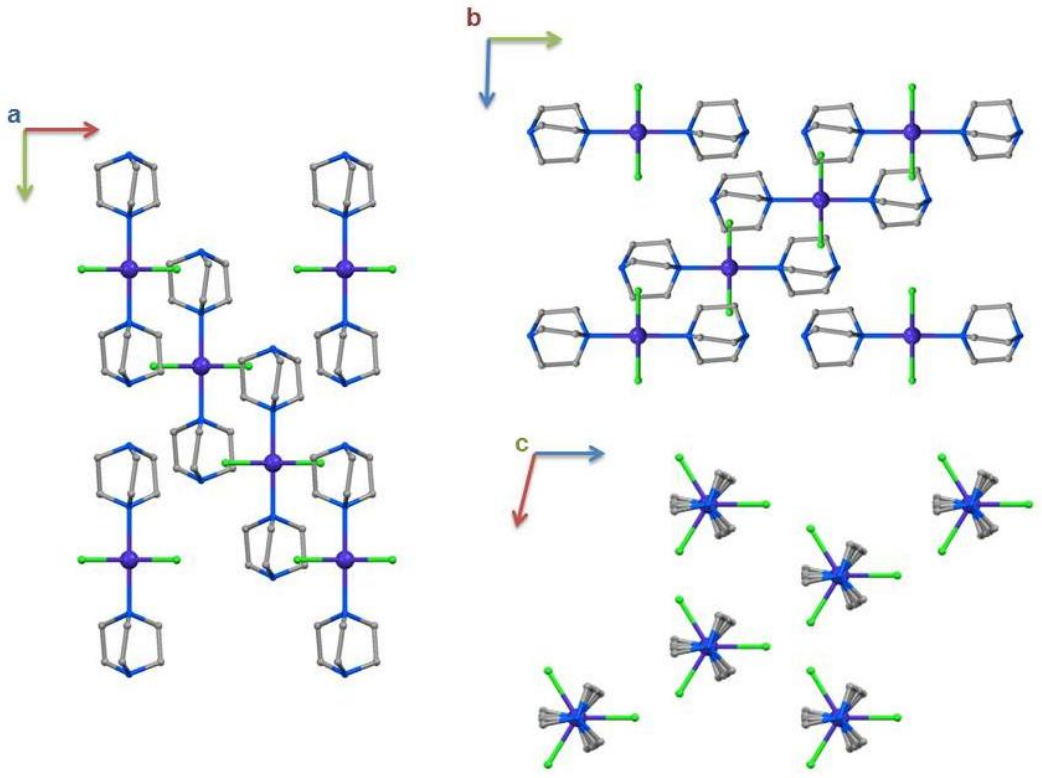

Table S2 Results from *SHAPE* studies[1–3] for complex **1** with the lowest CShM value highlighted in purple signifying the closest geometry of the complex.

		**CShM Values**
**Shape**	**Symmetry**	**1**
Pentagon	D_{5h}	37.138
Vacant Octahedron	C_{4v}	7.458
**Trigonal Bipyramid**	$\mathbf{D_{3h}}$	**0.015**
Spherical Square Pyramid	C_{4v}	5.389
Johnson Trigonal Bipyramid	D_{3h}	3.365

Figure S2 Depiction of the calculated powder X-ray diffraction (PXRD) of **1** based on single crystal XRD data collected at 100 K and the experimentally obtained powder X-ray diffraction collected at 290 K. The difference in intensities arises due to preferred orientation effects.

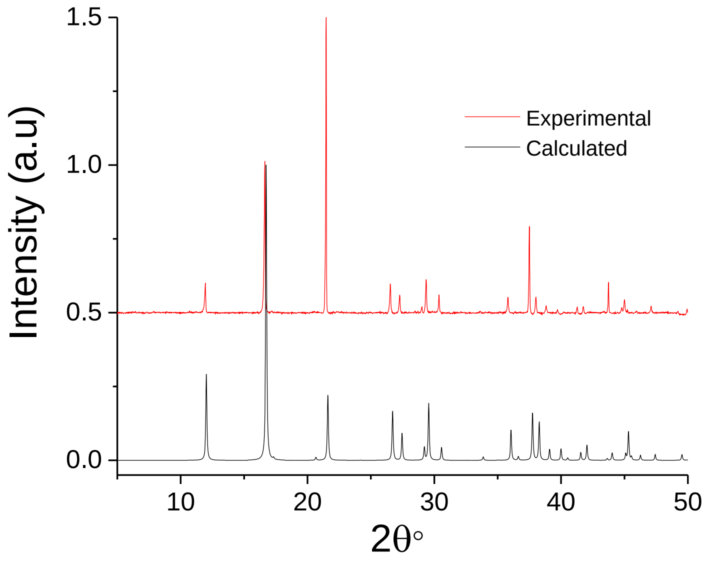

Table S3 NEVPT2 computed electronic states, the corresponding *major* electronic configurations and their individual contributions to the *D* and *E* values. Note that *minor* contributions are not shown and importantly, that the overall calculated *D* parameter comes from the diagonalisation of the whole *D* tensor rather than the summation of the contributions arising from individual states.

Energy (cm^{-1})	Electronic configurations from CASSCF	Contribution to *D* (cm^{-1})	Contribution to *E* (cm^{-1})
0 ($^4A_2'$)	$d_{xz}^2 d_{yz}^2 d_{x2-y2}^1 d_{xy}^1 d_{z2}^1$ (87%)	0.0	0.00
2620 ($^4A_1''$)	$d_{xz}^2 d_{yz}^1 d_{x2-y2}^1 d_{xy}^2 d_{z2}^1$ (50%)<br>$d_{xz}^1 d_{yz}^2 d_{x2-y2}^2 d_{xy}^1 d_{z2}^1$ (50%)	-0.03	0.00
2658 ($^4A_2''$)	$d_{xz}^2 d_{yz}^1 d_{x2-y2}^2 d_{xy}^1 d_{z2}^1$ (50%)<br>$d_{xz}^1 d_{yz}^2 d_{x2-y2}^1 d_{xy}^2 d_{z2}^1$ (50%)	0.00	0.00
4084 ($^4E''$)	$d_{xz}^2 d_{yz}^1 d_{x2-y2}^1 d_{xy}^2 d_{z2}^1$ (32%)<br>$d_{xz}^1 d_{yz}^2 d_{x2-y2}^2 d_{xy}^1 d_{z2}^1$ (32%)<br>$d_{xz}^1 d_{yz}^2 d_{x2-y2}^1 d_{xy}^1 d_{z2}^2$ (29%)	20.6	20.6
4087 ($^4E''$)	$d_{xz}^2 d_{yz}^1 d_{x2-y2}^2 d_{xy}^1 d_{z2}^1$ (32%)<br>$d_{xz}^1 d_{yz}^2 d_{x2-y2}^1 d_{xy}^2 d_{z2}^1$ (32%)<br>$d_{xz}^2 d_{yz}^1 d_{x2-y2}^1 d_{xy}^1 d_{z2}^2$ (29%)	20.6	-20.6
19616 ($^2A_1'$)	$d_{xz}^2 d_{yz}^2 d_{x2-y2}^0 d_{xy}^2 d_{z2}^1$ (32%)<br>$d_{xz}^2 d_{yz}^2 d_{x2-y2}^2 d_{xy}^0 d_{z2}^1$ (32%)	12.0	0.00

Figure S3 NEVPT2 computed quantitative quartet ligand field states of the molecule along with the D_{3h} point group terms.

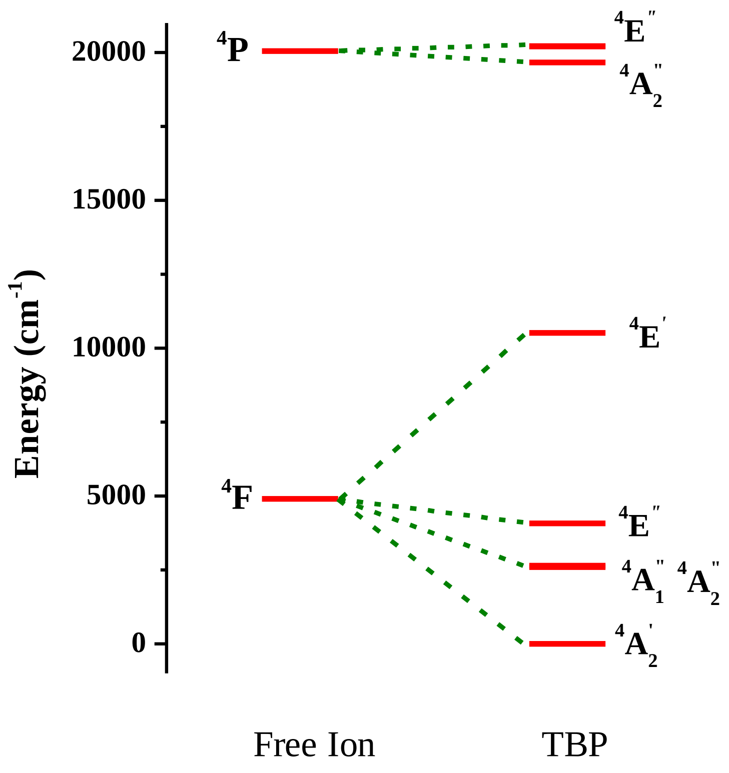

Figure S4 Representation of the components of the $^4A_2'$ ground state and the low energy $^4E''$ excited states, with the transition between these states providing the most significant contribution to D and E.

Ground ($^4A_2'$) state:

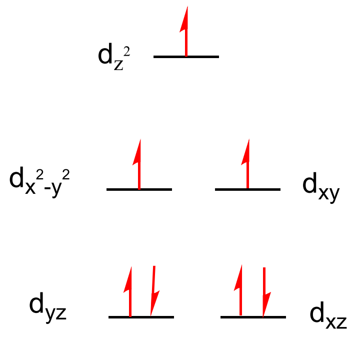

Components of excited $^4E''$ at 4084 cm^{-1}:

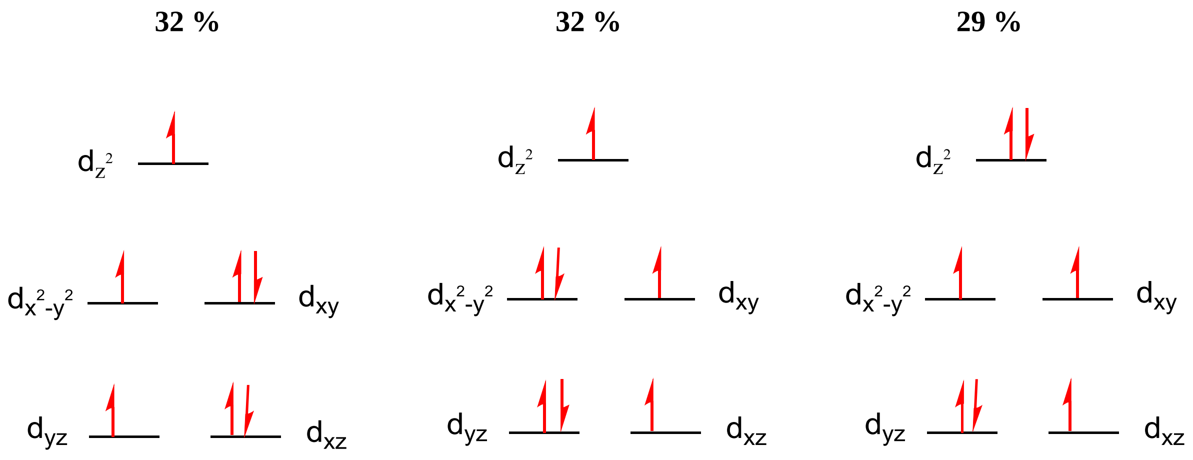

Components of excited ($^4E''$) state at 4087 cm^{-1}:

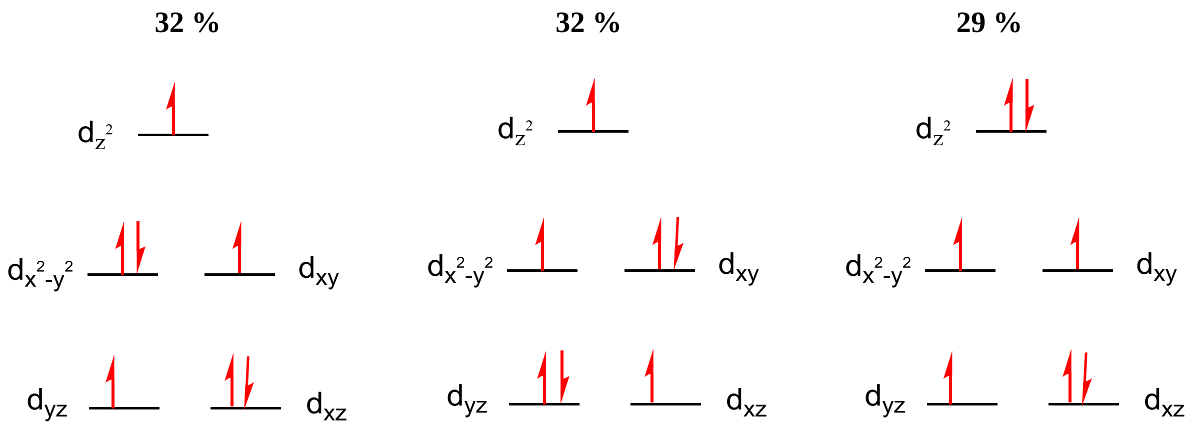

High-field EPR Details

High-field/frequency EPR spectra were collected on a microcrystalline powder sample of **1**, which was immobilized in a polyethylene cup with a Teflon® stopper. The transmission-type spectrometer used in this study employed a 17 T superconducting magnet.[4] Microwave frequencies were generated in the 50 to 635 GHz range using a phase-locked Virginia Diodes source combined with a series of frequency multipliers. The field modulated EPR signal, dI/dB (where I represents the absorption intensity and B the magnetic field strength), was obtained via lock-in detection using an InSb hot-electron bolometer (QMC Ltd., Cardiff, U.K.). Temperature control was achieved using an Oxford Instruments (Oxford, U.K.) continuous-flow cryostat.

Representative powder EPR spectra recorded at T = 5 K and multiple high frequencies in the range from 51.2 to 633.6 GHz are displayed in Fig. 3a (main text). On the basis of their relative

intensities and lineshapes, the two modes observed at the extremes of the spectra – one seen on the low field side as an asymmetric peak (marked with *) and the other on the high field side as a relatively symmetric dip (marked with #) – can be attributed to the perpendicular and parallel excitations within the lowest Kramers doublet, respectively, with effective Landé factors $g_{\perp}^{\text{eff}} = g_x^{\text{eff}} = g_y^{\text{eff}}$ = 4.80(2) and $g_{//}^{\text{eff}}$ = 2.03(1), as deduced from the plot of frequency versus resonance position in Fig. 3b (main text). Several sharper signals seen at fields slightly below the parallel excitation (see expanded views at 101.6, 201.6 and 319.2 GHz) are due to insufficient grinding of the powder sample, resulting in a few over-sized micro-crystals that give disproportionate responses above the continuum powder EPR spectrum. The extreme anisotropy of the *g*-tensor results in a very low density of spectral states at fields just below the parallel mode signal, which is why the spurious signals appear strongest in this range. This is compounded by the fact that the spectra were recorded in derivative mode, along with the extreme narrowness of the resonances arising from the individual crystallites (also an indication of exceptional sample quality, possibly due to the lack of solvent in the lattice). Several attempts were made to re-grind the sample, and the data displayed in Fig. 3a represent the best results.

Although it is not possible to constrain the zero-field splitting parameters *D* and *E* on the basis of EPR transitions associated with the lowest Kramers doublet, it is possible to constrain the sign of *D*. The observation of two g^{eff} values well above 2.00, and one near 2.00, is indicative of an easy-plane type anisotropy (positive *D* value for the *S* = 3/2 ground state). Moreover, the effective Landé factors associated with the lowest Kramers doublet may be related to the real *g*-tensor associated with the *S* = 3/2 multiplet through the following perturbative expressions[5,6]

$$g_x^{\text{eff}} = g_x\left(1 + \frac{1+3\gamma}{\sqrt{1+3\gamma^2}}\right);\;\; g_y^{\text{eff}} = g_y\left(1 + \frac{1-3\gamma}{\sqrt{1+3\gamma^2}}\right);\;\; g_z^{\text{eff}} = g_z\left(\frac{2}{\sqrt{1+3\gamma^2}} - 1\right), \quad \textbf{(S1)}$$

where $\gamma = E/D$. If one makes the reasonable assumption/approximation that *E*/*D* = 0 (see below), then,

$$g_x = g_y = \frac{g_{\perp}^{\text{eff}}}{2} = 2.40(1);\;\; g_z = g_z^{\text{eff}} = 2.03(1). \quad \textbf{(S2)}$$

Although it is not possible to discern any splitting of the perpendicular mode, it is noticeably broader than the parallel component. One possible explanation could be an unresolved splitting of the parallel mode due to a finite value of $\gamma = E/D$. From the peak-to-peak linewidth and the expressions in Eq. S1, it is possible to estimate an upper bound on *E*/*D* from the following expression:

$$\gamma \leq \frac{\delta g_{\perp}^{\text{eff}}}{3 g_{\perp}^{\text{eff}}} = 0.006, \quad \textbf{(S3)}$$

where $\delta g_{\perp}^{\text{eff}}$ is the spread in $g_{\perp}^{\text{eff}}$ corresponding to the peak-to-peak linewidth. Note that the theoretical upper bound on *E*/*D* lies well below this experimental upper bound. However, there could

be a multitude of other explanations for the perpendicular mode linewidth. In other words, to within the experimental resolution, the high-frequency EPR spectra of **1** appear to be quite axial.

Frequency Domain Magnetic Resonance Details

Frequency Domain Magnetic Resonance (FDMR) data were obtained by recording far-infrared (FIR) spectra under various external magnetic fields.[7] For this purpose a Bruker Vertex 80v vacuum Fourier transform infrared (FTIR) spectrometer with a resolution of 0.12 cm^{-1} was used. The sample was mounted in a 17 T superconducting magnet with optical access, such that the applied field was parallel to the direction of light propagation (Faraday geometry). The sample was in thermal equilibrium with the liquid Helium bath of the magnet and, therefore, at a temperature of 4.2 K. The transmitted FIR radiation was detected using a composite Si bolometer placed directly beneath the sample. Four FDMR spectra were recorded at each field between 0 and 17 T, in 1 T increments, in order to both average the spectra and evaluate the standard deviation (gray shading). To differentiate the magnetic from non-magnetic excitations (e.g. molecular vibrations and lattice phonons), each spectrum was divided by a reference. This procedure was performed for multiple choices of reference, all of which furnish final spectra with consistent field-dependent behavior. The divided spectra seen in Fig. 4 (main text) and Fig. S5 were prepared by using the spectrum recorded 4 T higher in applied field as the reference. The spectra displayed in Fig. S5 are identical to those in the main text, with the simulations removed in order to emphasize the raw magnetic excitations.

As can be seen in Fig. S5, the inter-Kramers transition is clearly observed at intermediate fields (3 to 10 T), but diminishes in intensity at the lowest fields. This may be due to overlap with strong non-magnetic absorption in the sample. To check for this, a thinner sample was separately investigated, and the obtained low-field spectra are shown in Fig. S6. Although the same intensity trend remains, the zero-field transition frequency can be clearly identified. In fact, two resonance branches are observed for this sample (see red shading), corresponding to allowed inter-Kramers excitations from each of the Zeeman split levels associated with the lowest Kramers doublet. The branch that moves to lower energies with increasing field involves an excitation from the higher-lying component of the doublet, thus explaining the reduction in intensity with increasing Zeeman splitting.

Figure S5 Normalized experimental FDMR spectra, identical to those in Fig. 4 (main text) with the simulations removed. The data were recorded at a temperature of 4.2 K, and the baseline of each spectrum is positioned according to the applied field strength on the ordinate. The gray shading provides a measure of the energy-dependent standard deviation of the FDMR signal from four separately recorded spectra. The blue and red shadings are guides to the eye, highlighting the intra- and inter-Kramers transitions, respectively.

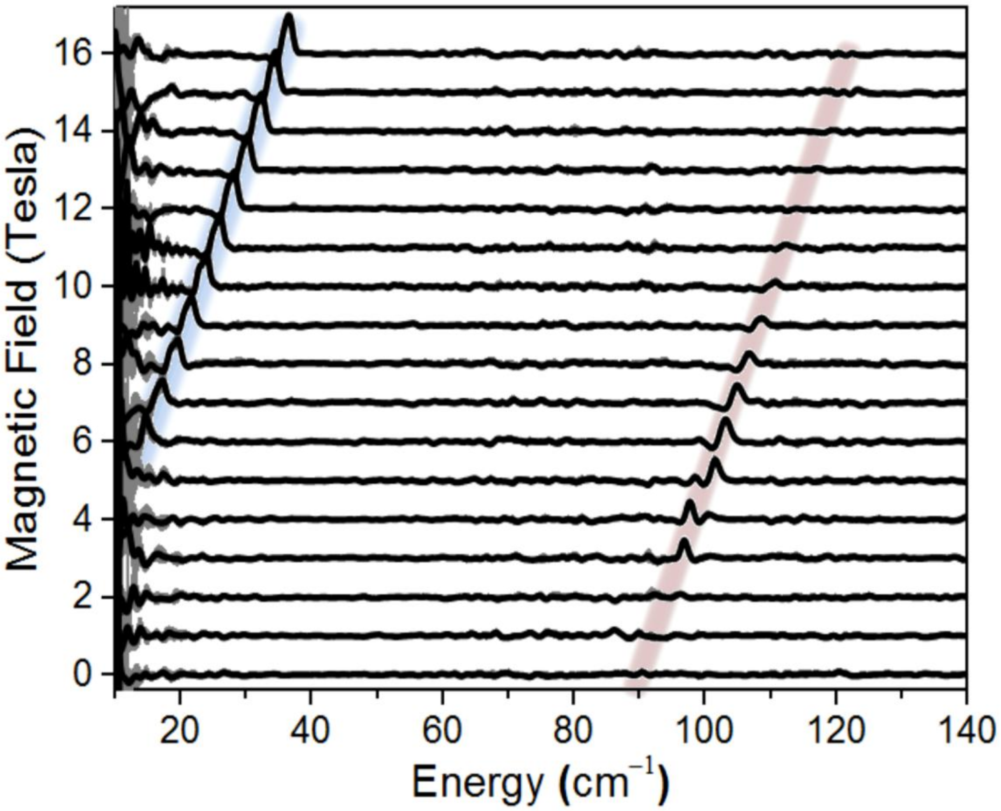

Figure S6 Normalized experimental FDMR spectra of a thinner sample. The data were recorded at a temperature of 4.2 K, and the baseline of each spectrum is positioned according to the applied field strength on the ordinate. The gray shading provides a measure of the energy-dependent standard deviation of the FDMR signal from four separately recorded spectra. The red shading is a guide to the eye, highlighting the allowed inter-Kramers transitions.

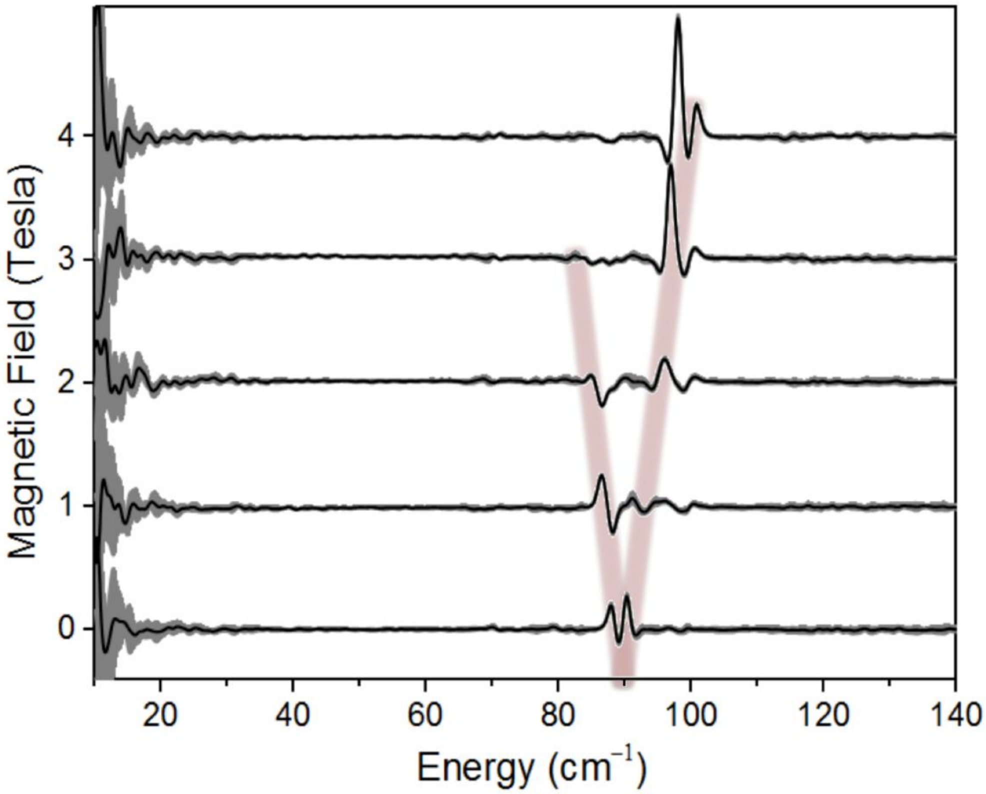

Figure S7 Variable-temperature magnetic susceptibility (1000 Oe, 290 K to 2 K) with the inverse magnetic susceptibility shown inset. The solid red line corresponds to the fit (see text for details).

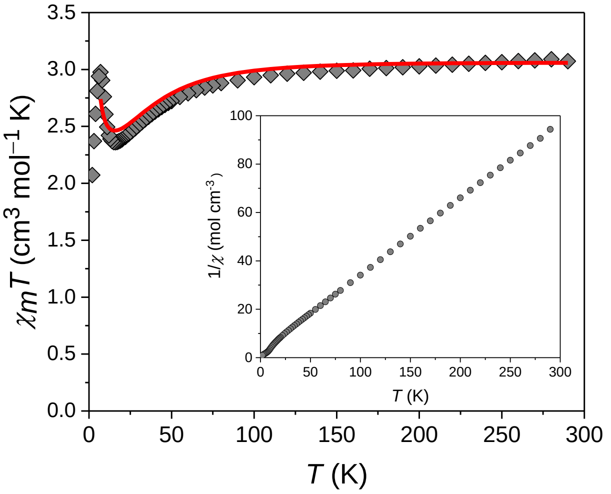

Fitting procedure for the dc magnetic data

Use of the full $\chi_m T$ vs T dataset proved unsuitable due to the low temperature maximum, which is consistent with competing weak ferro- and antiferro-magnetic short-range intermolecular interactions. Due to difficulties arising from simultaneously fitting two intermolecular interactions only the $\chi_m T$ *vs.* T data obtained above 6 K (290 - 7 K) were used for the fit, allowing us to successfully isolate (and reproduce the trend of) the upturn in $\chi_m T$ that is consistent with intermolecular ferromagnetic exchange interactions. This fit was performed using the program *Phi*,[8] according to the Hamiltonian presented in equation S4, where the first and second terms represent the axial zero-field splitting (ZFS) and Zeeman interactions, respectively (spin operator $\hat{S}$; applied field $\vec{B}$; Landé tensor *g*). The term relating to the contribution from intermolecular interactions (*zJ*) yielded a value of 0.27 cm^{-1}. The overall residual error was calculated in accordance with equation S5, yielding a value of 99.8 % indicating a good match between the experimental data and the fit.

$$\hat{H} = D\hat{S}_z^2 + \mu_B \vec{B} \cdot \overleftrightarrow{g} \cdot \hat{S} \qquad \textbf{(S4)}$$

$$Residual = [\sum_{i=1}^{points}(M_{exp} - M_{calc})^2][\sum_{i=1}^{points}(\chi_{exp} - \chi_{calc})^2] \qquad \textbf{(S5)}$$

Table S4 Previously reported zero-field splitting (ZFS) parameters for a selection of monometallic Co(II) complexes with a trigonal bipyramidal coordination environment. D_{theo} = value obtained via *ab initio* calculations; D_{exp} = value obtained via magnetic data (*) and HF-EPR (†). Easy-plane systems (+*D*) have been highlighted in blue for clarity.

	Denticity	Coord. Sphere	D_{theo} (cm^{-1})	D_{exp} (cm^{-1})	*E/D*
$[Co(Me_6tren)Cl)]\cdot ClO_4$[9]	Tetradentate	N_4Cl	-9.73	-8.12*†	-
$[Co(Me_6tren)Br]\cdot Br$[9]	Tetradentate	N_4Br	-2.12	-2.40*†	-
$[Co(TMPA)(CH_3CN)]\cdot BF_4$[10]	Tetradentate	N_5	+8.86	+9.66*	0.027
$[Co(TMPA)Cl]\cdot Cl$[10]	Tetradentate	N_4Cl	-8.63	-8.49*	-
$[Co(TMPA)Br]\cdot Br$[10]	Tetradentate	N_4Br	-5.30	-7.18*	-
$[Co(TMPA)I]\cdot I$[10]	Tetradentate	N_4I	-2.97	-7.53*	0.133
$[Co(tbta)N_3]\cdot(ClO_4)\cdot 3CH_3CN$[11]	Tetradentate	N_4N	-6.23	-10.7*	0.224
$[Co(NS_3^{iPr})Cl]\cdot(BPh_4)$[12]	Tetradentate	N_4Cl	-23.0	-19.9*†	0.075
$[Co(bpdmpz)Cl]\cdot PF_6$[13]	Tetradentate	N_4Cl	+4.52	+5.70*	0.243
$[Co(tpa)Cl]\cdot ClO_4$[14]	Tridentate	N_4Cl	-6.0	-10.1*	0.178
$[Co(tpa)Br]\cdot ClO_4$[14]	Tridentate	N_4Br	-4.4	-7.8*	0.269
$[Co(tbta)Cl]\cdot(ClO_4)\cdot(MeCN)_2\cdot(H_2O)$[14]	Tridentate	N_4Cl	-6.3	-7.5*	0.053
$[Co(tbta)Br]\cdot ClO_4$[14]	Tridentate	N_4Br	-4.4	-4.3*	0.0070
$[Co(bbp)(NCS)_2]$[15]	Tridentate	N_5	+8.44	+10.7*	0.00009
$[Co(phen)(DMSO)Cl_2$[16]	Bidentate	N_2OCl_2	-17.7	-17.0*†	0.240
$[(\gamma\text{-}CD)_2Co_4Li(H_2O)_{12}]$[17]	Bidentate	O_5	-	+27.9*†	0.226
$[Co^{II}Co_2^{III}(\mu_3\text{-}OH)(\mu\text{-}pz)_4(DBM)_3]\cdot 2MeCN$[18]	Bidentate	N_2O_3	-	+23.85*	0.170
$[Co(PDA)(H_2O)_2]$[19]	Monodentate	N_1O_4	-	+16.0*	0.125
$[Co(PDA)(1,2\text{-}BIYB)_{0.5}(H_2O)]$[19]	Monodentate	N_2O_3	-	+59.0*	0.119
**$[CoCl_3(HDABCO)(DABCO)]$ this work**	**Monodentate**	**N_2Cl_3**	**+44.18**	**+44.50*†**	**0**

Me_6tren = Tris[2-(dimethylamino)ethyl]amine; TMPA = tris(2-pyridylmethyl)amine; tbta = tris[(1- benzyl-1H-1,2,3-triazol-4-yl)methyl]amine); NS_3^{iPr} = 2-(isopropylthio)ethyl)- amine; bpdmpz = bis[(2-pyridylmethyl)-(di(3,5-dimethyl-1H-pyrazolyl)methyl)]amine; tpa = tris(2-methylpyridyl)amine; bbp = 2,6-bis(2- benzimidazolyl)pyridine; phen = 1,10-phenanthroline; γ-CD = γ-cyclodextrin; H_2PDA = pyridine-3,5-dicarboxylate; 1,2-BIYB = 1,2-BIYB = 1,2-bis(imidazol-1-ylmethyl) benzene).

Figure S8 Frequency-dependence of the ac susceptibility between 0 and 5000 Oe at 2 K with the in-phase χ'_m and out-of-phase χ''_m signals shown above and below, respectively.

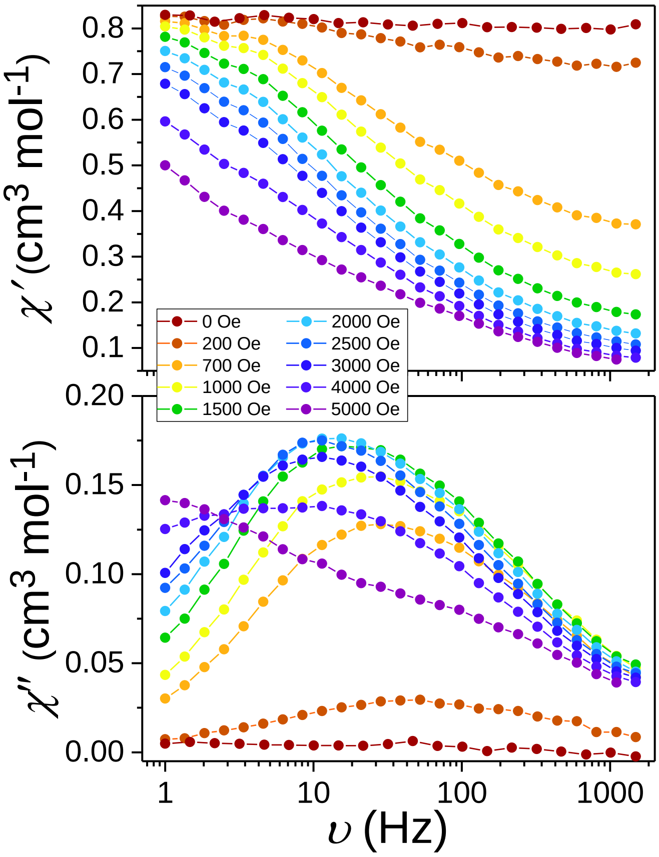

Figure S9 Argand diagram (χ' vs. χ'') for fields between 200 and 4000 Oe at 2 K.[20,21] Data collected at 0 and 5000 Oe have been omitted for clarity.

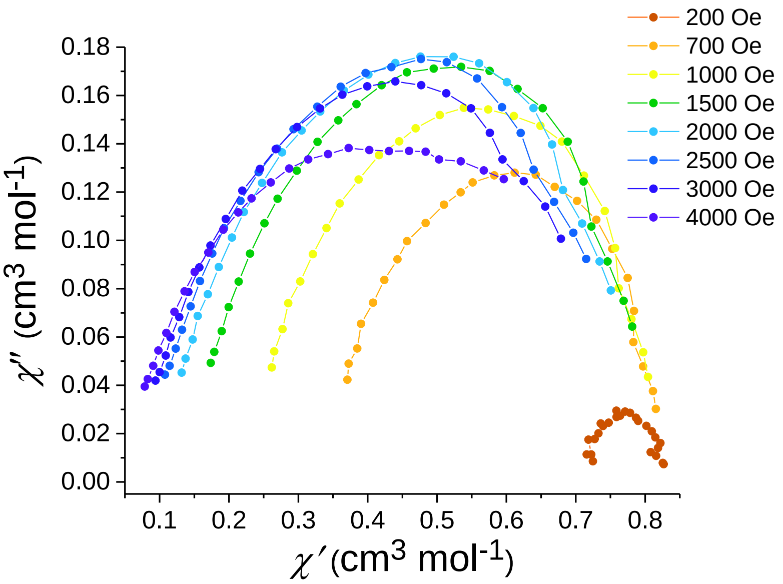

Figure S10 Field dependence of the maximum in the out-of-phase (χ'') response and the corresponding frequency at which it is observed for fields between 200 and 4000 Oe at 2 K. The maximum χ'' response is at 2500 Oe.

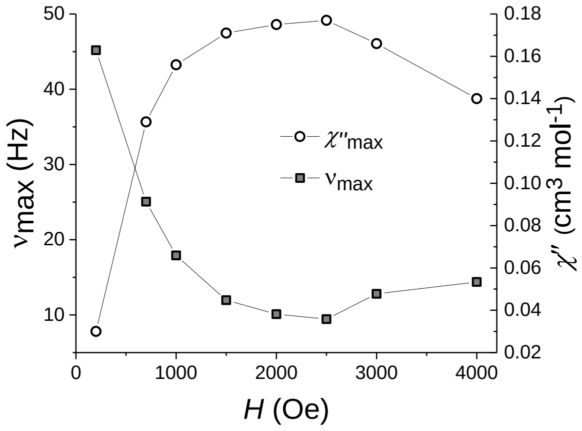

Figure S11 Argand plots (χ''_m vs. χ'_m) from which the temperature-dependence of the relaxation rates is obtained between 2.4 and 7 K under an applied field of 2500 Oe. Solid lines correspond to fits of the data.[20,21]

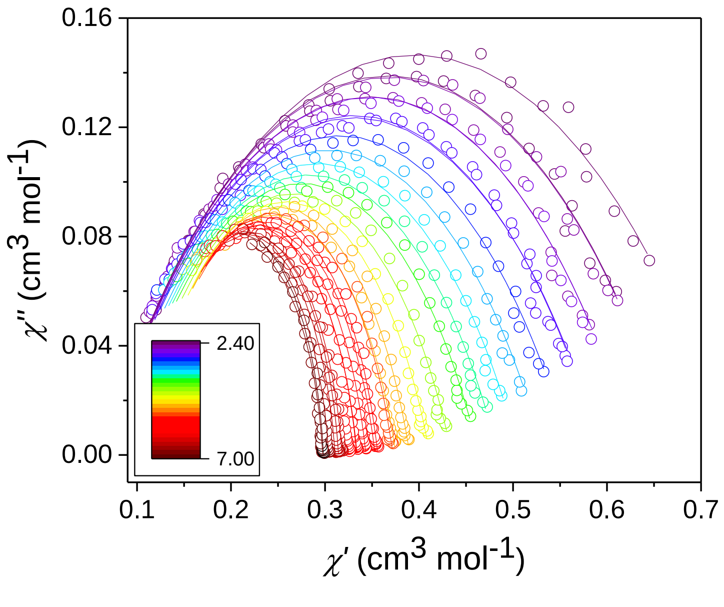

Figure S12 Field dependence of the inverse of the relaxation rates (1/τ) for fields between 200 – 4000 Oe at 2 K where the solid red line corresponds to the fit (see main text for details).

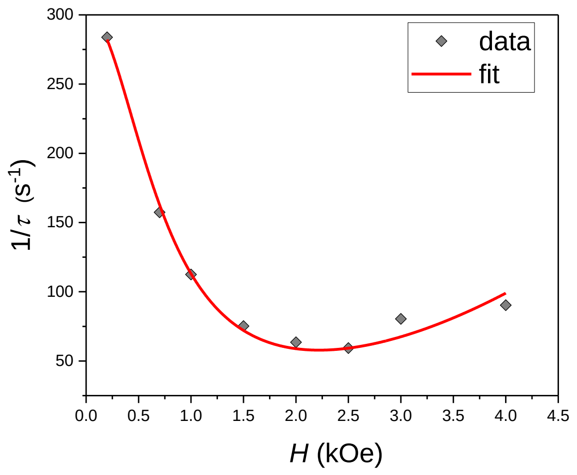

$$\tau^{-1} = AH^2T + \frac{B_1}{1+B_2H^2} \qquad \text{(S6)}$$

Fitting procedure for the temperature dependent ac magnetic data

An Argand plot (χ' vs. χ'') can be used to quantify the relaxation time (τ) and its distribution ($0 \leq \alpha \leq 1$) with appropriate values extracted from a fit of this data using the program *CC-Fit*.[20,21] At a fixed field and temperature, the ac susceptibility can be described by the following expression[20]:

$$\chi_{AC}(\omega) = \chi_s + \frac{\chi_T - \chi_S}{1+(i\omega\tau)^{(1-\alpha)}} \qquad \text{(S7)}$$

The terms χ_s and χ_T are the adiabatic and isothermal susceptibilities respectively, with ω denoting the angular frequency of the oscillating field. Where relaxation of magnetisation occurs with a single Debye Process, or relaxation time, Debye theory predicts the Argand diagram will resemble a single semi-circular arch ($\alpha = 0$).[20,21] When more than one time constant is present ($0 < \alpha < 1$), the distribution of time constants flattens the observed semi-circle.[20]

The temperature-dependent ac susceptibility data were best fitted to equation S7 with only a single semi-circular arch in the Argand plot. The α parameter indicates multiple relaxation times in the lower temperature region between 2.4 and 4.6 K ($0.27 < \alpha < 0.57$). At higher temperatures, the distribution of relaxation times is less broad with $0.08 < \alpha < 0.27$.

Table S5 Cole-Cole fit value for **1** at an applied dc field of 2500 Oe between 2.4 and 7.0 K.

T (K)	χ_S(cm^3 mol^{-1})	χ_T (cm^3 mol^{-1})	τ (s)	α	Residual
2.4	0.06186	0.73028	0.00713	0.47393	7.80334E-4
2.5	0.06378	0.67233	0.00538	0.45471	9.4798E-4
2.6	0.06438	0.67205	0.00501	0.456	7.84389E-4
2.7	0.06618	0.63047	0.00412	0.44563	8.18632E-4
2.8	0.06644	0.63062	0.00381	0.44653	7.7213E-4
2.9	0.06902	0.59337	0.00321	0.43616	9.70828E-4
3.0	0.06757	0.59508	0.00297	0.44234	7.71957E-4
3.2	0.0693	0.56216	0.00236	0.43589	6.88007E-4
3.4	0.07441	0.53305	0.00192	0.4231	6.13145E-4
3.6	0.07844	0.50683	0.00157	0.41132	5.97794E-4
3.8	0.08202	0.48431	0.00129	0.39944	6.35734E-4
4.0	0.08722	0.46369	0.00107	0.38108	6.03946E-4
4.3	0.09541	0.43496	8.1469E-4	0.34658	6.21102E-4
4.6	0.10971	0.39024	4.88477E-4	0.26742	5.25884E-4
4.9	0.11056	0.37551	4.43001E-4	0.25327	4.94163E-4
5.0	0.11548	0.37227	3.78197E-4	0.23049	4.3512E-4
5.2	0.11484	0.3533	3.05746E-4	0.20684	3.05443E-4
5.4	0.11829	0.3562	2.88396E-4	0.19392	2.90513E-4
5.5	0.11857	0.34352	2.63039E-4	0.18069	2.39897E-4
5.6	0.11973	0.33418	2.19501E-4	0.15888	1.24447E-4
5.8	0.11931	0.33355	1.84032E-4	0.15044	1.51196E-4
6.0	0.12127	0.31808	1.55741E-4	0.12243	7.82352E-5
6.2	0.11374	0.31068	1.23866E-4	0.11719	6.22142E-5
6.4	0.11849	0.31433	1.17045E-4	0.11076	6.41167E-5
6.5	0.10971	0.30387	1.01498E-4	0.11103	4.94678E-5
6.6	0.11298	0.29748	8.70228E-5	0.09873	3.24E-5
6.8	0.11021	0.29828	7.21143E-5	0.08404	4.51691E-5
7.0	0.10971	0.39024	4.88477E-4	0.26742	5.25884E-4

When relaxation occurs *via* an Orbach process, a linear approximation of $\ln\tau$ *vs.* T^{-1} can be made to determine the activation energy (ΔE) in accordance with the Arrhenius Law, as shown in equation S8.[22] A straight line approximation using data collected in the higher temperature region between 4.9 K and 7.0 K was performed yielding values of $\Delta E/k$ = 31.00 ± 1.02 K (21.5 cm^{-1}) and τ_0 = 9.69 ± 0.18 x 10^{-7} s (see Fig. S12). The value for $\Delta E/k$ is much lower than that based on the D value from FDMR and *ab initio* calculations ($\Delta E = |2D| \sim 89$ cm^{-1}).

$$\tau^{-1} = \tau_0^{-1} \exp\left(\frac{-\Delta E}{kT}\right) \quad \textbf{(S8)}$$

Figure S13 Arrhenius plot (ln τ vs. 1/T) of the temperature-dependence of the relaxation rates between 2.4 and 7 K under H_{dc} = 2500 Oe. The solid red line corresponds to the linear fit to equation S8 of the high temperature (4.9 – 7 K) data giving an activation energy $\Delta E/k$ = 31.00 ± 1.02 K (21.5 cm^{-1}) and τ_0 = 9.69 x 10^{-7} s. If one were to associate this activation energy with an Orbach process, the experimentally determined activation energy is much lower than the zero-field splitting found between the m_s = ± 1/2 and m_s = ±3/2 states by the FDMR measurements (|2D_{mag}| = ~21.5 cm^{-1} *vs.* |2D_{FDMR}| = ~89 cm^{-1}).

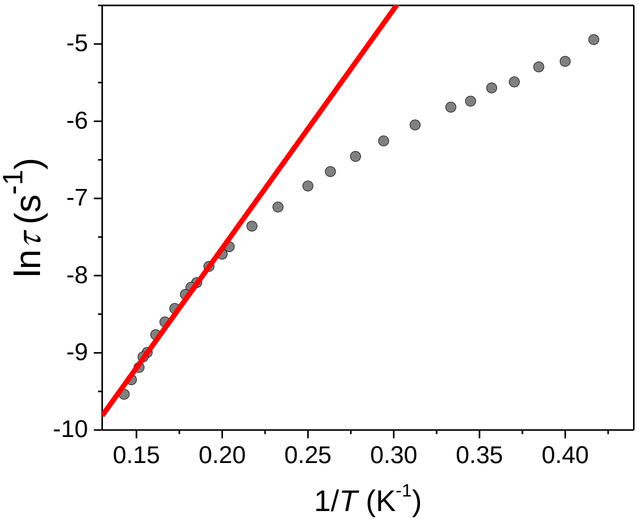

Table S6 Parameters for direct and QTM relaxation processes (obtained via the fit of the field dependence of the relaxation rates in accordance with equation S6) and for the Raman process (obtained via the fit of the temperature dependence of the relaxation rates in accordance with equation 1 in the main text) for complex **1**.

A **(s^{-1} Oe^{-2} K^{-1})**	***B*$_1$ (s^{-1})**	***B*$_2$ (Oe^{-2})**	***C*** **(s^{-1} K^{-n})**	***n***
277.9 (20.9)	302.4 (9.5)	181.2 (17.2)	0.20(0.04)	5.70 (0.09)

References

(1) M. Pinsky and D. Avnir, *Inorg. Chem.* 1998, *37*, 5575.

(2) D. Casanova, J, Cirera, M. Llunell, P. Alemany, D. Avnir and S. Alvarez, *J. Am. Chem. Soc.* 2004, *126*, 1755.

(3) S. Alvarez, *Chem. Rev.* 2015, *115*, 13447.

(4) A. K. Hassan, L. A. Pardi, J. Krzystek, A. Sienkiewicz, P. Goy, M. Rohrer and L. C. Brunel, Ultrawide Band Multifrequency High-Field EMR Technique: A Methodology for Increasing Spectroscopic Information. *J. Magn. Reson.* 2000, *142*, 300.

(5) T. Yamane, K. Sugisaki, T. Nakagawa, H. Matsuoka, T. Nishio, S. Kinjyo, N. Mori, S. Yokoyama, C. Kawashima, N. Yokokura et al. *Phys. Chem. Chem. Phys.* 2017, *19,* 24769.

(6) T. Yamane, K. Sugisaki, H. Matsuoka, K. Sato, K. Toyota, D. Shiomi, T. Takui, *Dalton Trans.* 2018, *47,* 16429.

(7) J. Ludwig, Y. B. Vasilyev, N. N. Mikhailov, J. M. Poumirol, Z. Jiang, O. Vafek and D. Smirnov, *Phys. Rev. B* 2014, *89*, 241406.

(8) N. F. Chilton, R. P. Anderson, L. D. Turner, A. Soncini and K. S. Murray, *J. Comput. Chem.* 2013, *34*, 1164.

(9) R. Ruamps, L. J. Batchelor, R. Guillot, G. Zakhia, A.-L. Barra, W. Wernsdorfer, N. Guihéry and T. Mallah, *Chem. Sci.* 2014, *5*, 3418.

(10) T. J. Woods, M. F. Ballesteros-Rivas, S. Gómez-Coca, E. Ruiz and K. R. Dunbar, *J. Am. Chem. Soc.* 2016, *138*, 16407.

(11) D. Schweinfurth, M. G. Sommer, M. Atanasov, S. Demeshko, S. Hohloch, F. Meyer, F. Neese and B. Sarkar, *J. Am. Chem. Soc.* 2015, *137*, 1993.

(12) F. Shao, B. Cahier, N. Guihery, E. Riviere, R. Guillot, A.-L. Barra, Y. Lan, W. Wernsdorfer, V. E. Campbell and T. Mallah, *Chem. Commun.* 2015, *51*, 16475.

(13) S. S. Massoud, R. C. Fischer, F. A. Mautner, M. M. Parfait, R. Herchel and Z. Trávníček, *Inorganica Chim. Acta* 2018, *471*, 630.

(14) A. K. Mondal, J. Jover, E. Ruiz and S. Konar, *Chem. - A Eur. J.* 2017, *23*, 12550.

(15) A. K. Mondal, T. Goswami, A. Misra and S. Konar *Inorg. Chem.* 2017, *56*, 6870.

(16) I. Nemec, R. Marx, R. Herchel, P. Neugebauer, J. van Slageren and Z. Travnicek, *Dalton Trans.* 2015, *44*, 15014.

(17) N. Nedelko, A. Kornowicz, I. Justyniak, P. Aleshkevych, D. Prochowicz, P. Krupiński, O. Dorosh, A. Ślawska-Waniewska and J. Lewiński, *Inorg. Chem.* 2014, *53*, 12870.

(18) A. Collet, G. A. Craig, M. J. Heras Ojea, L. Bhaskaran, C. Wilson, S. Hill and M. Murrie, *Dalton Trans.* 2018, *47*, 9237.

(19) X. Hou, X. Wang, X. Liu, J.-J. Wang, L. Tang and P. Ju, *New J. Chem.* 2018, *42*, 8583.

(20) Y.-N. Guo, G.-F. Xu, Y. Guo and J. Tang, *Dalton Trans.* 2011, *40* , 9953.

(21) K. S. Cole and R. H. Cole, *J. Chem. Phys.* 1941, *9*, 341.

(22) R. L. Carlin, *Magnetochemistry*; Springer-Verlag: Berlin, 1986.

(23) S. T. Liddle and J. van Slageren, *Chem. Soc. Rev.* 2015, *44*, 6655.

(24) V. V. Novikov, A. A. Pavlov, Y. V. Nelyubina, M.-E. Boulon, O. A. Varzatskii, Y. Z. Voloshin and R. E. P. Winpenny, *J. Am. Chem. Soc.* 2015, *137*, 9792.

(25) C. Rajnák J. Titiš, J. Moncoľ, F. Renz and R. Boča, *Eur. J. Inorg. Chem.* 2017, 1520.

(26) K. Shrivastava, *Phys. Status Solidi B* 1983, *117*, 437.

(27) A. Singh and K. Shrivastava, *Phys. Status Solidi B* 1979, *95*, 273.

(28) G. A. Santillan, C. J. Carrano, *Inorg. Chem.* 2007, *46*, 1751.
